# A Model-Based Approach to Neuronal Electrical Activity and Spatial Organization Through the Neuronal Actin Cytoskeleton

**DOI:** 10.3390/mps8040076

**Published:** 2025-07-07

**Authors:** Ali H. Rafati, Sâmia Joca, Regina T. Vontell, Carina Mallard, Gregers Wegener, Maryam Ardalan

**Affiliations:** 1 Translational Neuropsychiatry Unit, Department of Clinical Medicine, Aarhus University, 8200 Aarhus, Denmark; sjoca@biomed.au.dk (S.J.); wegener@clin.au.dk (G.W.); 2 Center of Functionally Integrative Neuroscience-SKS, Department of Clinical Medicine, Aarhus University, 8200 Aarhus, Denmark; 3 Department of Biomedicine—Forskning og Uddannelse, Vest, Aarhus University, 8200 Aarhus, Denmark; 4 Department of Neurology, University of Miami Miller School of Medicine, Brain Endowment Bank, Miami, FL 00650, USA; rvontell@miami.edu; 5 Institute of Neuroscience and Physiology, Department of Physiology, Sahlgrenska Academy, University of Gothenburg, 40530 Gothenburg, Sweden; carina.mallard@gu.se

**Keywords:** actin cytoskeleton, action potential, mathematical modeling, neuronal signaling, organoid brain, primary cilia

## Abstract

The study of neuronal electrical activity and spatial organization is essential for uncovering the mechanisms that regulate neuronal electrophysiology and function. Mathematical models have been utilized to analyze the structural properties of neuronal networks, predict connectivity patterns, and examine how morphological changes impact neural network function. In this study, we aimed to explore the role of the actin cytoskeleton in neuronal signaling via primary cilia and to elucidate the role of the actin network in conjunction with neuronal electrical activity in shaping spatial neuronal formation and organization, as demonstrated by relevant mathematical models. Our proposed model is based on the polygamma function, a mathematical application of ramification, and a geometrical definition of the actin cytoskeleton via complex numbers, ring polynomials, homogeneous polynomials, characteristic polynomials, gradients, the Dirac delta function, the vector Laplacian, the Goldman equation, and the Lie bracket of vector fields. We were able to reflect the effects of neuronal electrical activity, as modeled by the Van der Pol equation in combination with the actin cytoskeleton, on neuronal morphology in a 2D model. In the next step, we converted the 2D model into a 3D model of neuronal electrical activity, known as a core-shell model, in which our generated membrane potential is compatible with the neuronal membrane potential (in millivolts, mV). The generated neurons can grow and develop like an organoid brain based on the developed mathematical equations. Furthermore, we mathematically introduced the signal transduction of primary cilia in neurons. Additionally, we proposed a geometrical model of the neuronal branching pattern, which we described as ramification, that could serve as an alternative mathematical explanation for the branching pattern emanating from the neuronal soma. In conclusion, we highlighted the relationship between the actin cytoskeleton and the signaling processes of primary cilia. We also developed a 3D model that integrates the geometric organization unique to neurons, which contains soma and branches, such that the mathematical model represents the interaction between the actin cytoskeleton and neuronal electrical activity in generating action potentials. Next, we could generalize the model into a cluster of neurons, similar to an organoid brain model. This mathematical framework offers promising applications in artificial intelligence and advancements in neural networks.

## 1. Introduction

It is believed that a better understanding of brain functions at the molecular and cellular levels could be beneficial in developing an advanced artificial neural network with high potential for being transformed into a computational model with a capacity comparable to that of the human brain. However, the prerequisite is to explore the neurobiological systems deeply and then translate them mathematically. Neuronal connectivity is a function of neuronal branches, including dendrites and axons, that converge in a crosstalk model between neurons, astrocytes, and microglia [1]. Neuronal connections occur through spontaneous neuronal activity, and synapse formation and elimination are highly dynamic; thus, appropriate synaptic formation is dependent on neuronal activity [2,3]. Furthermore, actin is a cytoskeletal protein that contributes to neuronal polarity, the movement of growth cones, and aggregation in the initial segment and the whole axon; it is involved in the plasticity of dendritic spines, dendrite morphology and primary cilia, and its principal functions are cell shape formation, migration, and intracellular trafficking [4]. The actin cytoskeleton is believed to mediate spine morphogenesis as part of synapses, as it forms networks in the spine to support its structure. Neurotransmitter-mediated signaling through spines regulates neurodevelopmental processes through spontaneous Ca^2+^ activity. Spontaneous Ca^2+^ oscillations via gap junctions play an essential role during embryonic neurodevelopment, in which co-induction by Sonic Hedgehog signaling (Shh) regulates the Ca^2+^ spike [5]. Mathematical models can help elucidate how ion channel properties influence neuronal excitability and contribute to the generation and propagation of action potentials.

Additionally, the actin cytoskeleton is controlled by incoming signals via a secreted extracellular matrix glycoprotein called Reelin, which modulates dendrite arborization during the neurodevelopmental period [6]. Therefore, the dendritic actin cytoskeleton is modified by external signals to control synaptic function [6,7], and actin morphology is influenced by neural cell adhesion molecule-1 (NCAM-1), which contributes to neuronal migration and dendritic arborization [8]. The other function of actin is related to neuronal primary cilia [9], which are protrusions from the neuronal surface containing the actin cytoskeleton and microtubule-based appendages, and function as antennas for signals [10]. During embryonic development, cilia play a crucial role in cell migration, signal transduction, and Hedgehog signaling [11], which ultimately influences neurodevelopment. Therefore, the roles of primary cilia, globular actin (G-actin), and F-actin (filamentous actin) [12,13] in synaptic formation and normal brain development have been explored. Studying neuronal shape and electrical activity is expected to shed light on the underlying mechanisms that modulate neuronal electrophysiology and function, particularly in relation to the cellular cytoskeleton and morphology. Mathematical models can aid in understanding the kinetics of neurotransmitter release and reuptake and how these processes are modulated under different conditions and can be used to analyze the structural properties of neuronal networks, predict connectivity patterns, and investigate how alterations in morphology impact neural function.

Overall, experimental studies and observational data can provide valuable insights into these mechanisms. However, mathematical modeling can offer advantages such as simulating hypothetical scenarios, predicting outcomes, and uncovering underlying principles that may not be immediately apparent from experimental data alone. Therefore, while mathematical modeling is not always indispensable, it can be a valuable tool in advancing our understanding of the role of the actin cytoskeleton in neuronal signaling [14,15,16]. This modeling can help in understanding the complex dynamics of neuronal networks and provide insights into the mechanisms underlying synaptic plasticity, as well as its contribution to neural circuit function.

We designed this study as a complement to our previous study, which focused on a general mathematical model of neurodevelopment [17]. However, in this study, we aimed to explore the special mathematical equations that verify the interaction of the actin cytoskeleton with neuronal electrical activity, which is spatially organized and forms a 3D neuron with electrical properties that can grow and develop into a network of neurons. Furthermore, geometrical structures such as neuronal branches and signal transduction through primary cilia are also explored as affecting the dynamic regulation of actin, independent of the stage of neuronal organization.

## 2. Method

In this study, we applied essential mathematical equations and algorithms. The written equations in the form of functions were implemented in MATLAB (R2021B) to generate the plots. The LaTeX function in MATLAB was used to display the formula shown. Biorender (https://app.biorender.com/ (accessed on 15 December 2024)) was used to generate schematic illustrations. We proposed a mathematical definition of branching pattern that refers to the ramification from a point [18], as explained by Vandermonde polynomials [19]. Hence, in a more complex scenario, we proposed a mathematical model that illustrates how neuronal branches, including axons and dendrites, ramify from the neuronal soma during neurodevelopment, leading to synapse formation. Thus, the Möbius transformation and the rational function were used to model the geometry of branching from the soma in a topologically constrained 3D space. To generate the circles and spheres that represent the cytoskeleton of the neuronal soma, we applied the ring polynomial [20] and homogenous polynomial [21] in connection with the characteristic polynomial [22].

Furthermore, we introduced another mathematical model that applied vector fields and Maxwell’s equations in connection with the geometrical structure of the neuronal soma and branches, which are built by the actin cytoskeleton, the main contributor to the neuronal soma’s structure. The vector fields represent spatially varying quantities such as electric or membrane potential across neuronal structures. In neurobiology, the current flow, or morphological influences such as actin cytoskeleton formation in interaction with ion channels, can be visualized using vector fields. For example, the interactions of Kv3 ion channels with the actin cytoskeleton have been shown to contribute to neuronal shape configuration and change [23]. Moreover, the Lie brackets [24] measure the interaction between two vector fields, capturing how one field modifies the flow of another, which is useful for modeling nonlinear interactions, such as ion channel-cytoskeleton coupling or signal propagation influenced by cellular geometry.

The neurobiological reason for using the actin cytoskeleton is discussed in Section 2. A different application of the van der Pol oscillator [25] was incorporated to model the electrical activity in the form of the action potential and membrane potential of neurons, which is a novel representation of the 3D shape of a neuron with electrical properties similar to the physiological membrane potential. The processes of neuronal shape change and neuronal branching formation were introduced through our model, based on Frobenius’ theorem and the Lie bracket of vector fields. Finally, the model was completed by applying calculations related to the Goldman equation [26], the Nernst equation [27] to approximate ion-based potentials under simplified assumptions, and the Dirac delta function [28] to model the ionic inputs affecting the membrane potential of neurons [29]. In the last step, the electric field (E=−∇F, which means the gradient of the potential) was applied to estimate spatially varying potential fields on the modeled neuron surface, and the vector Laplacian was calculated [30] comparatively. Furthermore, the method for generating a 3D neural network involves a series of mathematical equations to guide neuron growth and interaction spatially. The process begins with the vector field interaction, which is given by Equation (12), that is, “V”, and represents the interaction between actin filaments and van der Pol oscillators. Next, the guiding $P(x,y,z)=-x^{3}z^{3}-ax^{2}z+x+y^{2}z^{2}-b$ that represents a multivariate polynomial constructed to resemble the structure of an elliptic curve $y^{2}=x^{3}+a\;x+b$ on a finite field [31,32] such that it introduces curvature that affects the growth of neurons. Next, the partial derivatives of P(x, y, z) are added to “V” to determine the directional influence on the neurons. The cumulative effects of these are calculated through integration, resulting in $\;G_{x},\;G_{y},\;G_{z}$. To guide the spatial placement of neurons, Lagrange interpolation is applied using the inverses of the Vandermonde matrix [33].

## 3. Results

### 3.1. The Role of Primary Cilia and Actin Cytoskeleton in Signal Transduction Through the Cell Membrane

**Figure 1:**
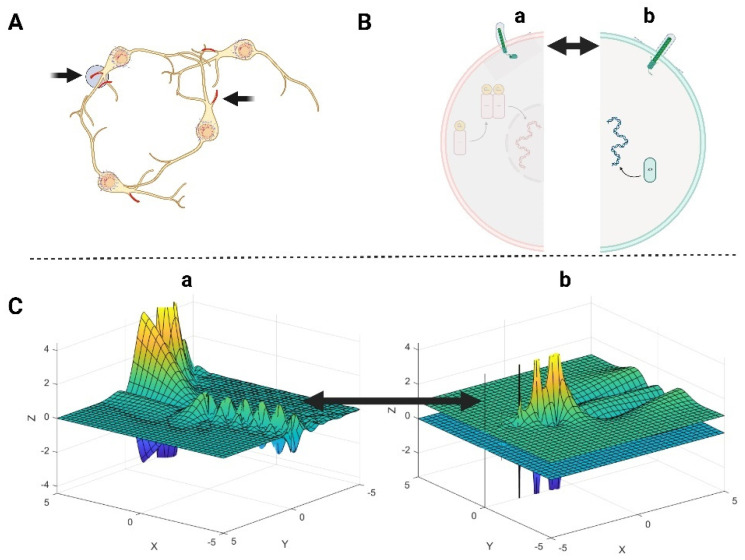
The schematic illustrations of neuronal primary cilia and the suggested signaling equation. (**A**) Neuronal primary cilia are indicated by black arrows that receive signals from regional neuronal networks. (**B**) Schematic illustration showing the signaling in the form of Sonic Hedgehog (Shh) via primary cilia; (**C**) The (a) and (b) denote two shapes of signaling that differ based on the calculation of complex number in the given incomplete Gamma function, $Q\left(x+iy,z\right)$ and P(x+iy,z) z=−5:0., x, y are spatial coordinate. The color coding in panel C of the figure (the 3D plots labeled “a” and “b”) represents the magnitude of the output of the signaling equation involving the incomplete Gamma function. Yellow Peaks: indicate highest magnitude or strongest signal intensity. Green color: represent moderate signal strength. Blue/Purple color: show low or negative signal intensity.

The Primary Cilium is a protrusion characterized by a microtubule structure in its core and a mesh of actin and actin-binding proteins located beneath the ciliary membrane. This actin network also exists at the base of the cilia structure, in proximity to the cell membrane [13]. The primary cilium senses biomechanical signals and changes in bending state, which are essential for relaying signals. Specifically, the primary cilia mediate Sonic Hedgehog (Shh) signaling [34], which is involved in the cell proliferation of neural precursors and stem cells during embryonic CNS development. The Shh is regulated by a mechanism involving the aggregation of proteoglycans and lipoproteins on the cell surface, indicating a relationship between the distribution of these aggregates and Shh diffusion. Thus, the formation of these aggregates was explained by the gamma function, and it was shown that the aggregates have a gamma distribution with right skewness [35]. The incomplete Gamma function $L\left(x,y,z\right)=Q(x+iy,z)=\left(1-\frac{\gamma (x+yi,z)}{\Gamma (x+yi)}\right)$, $U\left(x,y,z\right)=P(x+iy,z)=\left(\frac{\gamma (x+yi,z)}{\Gamma (x+yi)}\right)$ is applied into our model to represent the partial accumulation of Shh aggregates over time and space, which is a crucial feature in understanding Shh’s role in signaling via the primary cilium. The schematic illustration of primary cilia is demonstrated in Figure 1, which includes a mathematical equation containing the gamma and incomplete gamma functions [36] that represent an equivalence to the Sonic Hedgehog (Shh) signaling in primary cilia, showing Shh diffusion in 3D space. The suggested mathematical equation, shown in Figure 1, resembles fractional calculus-based models. The fractional differential equations, such as the Caputo fractional derivative $D_{x}^{m+\alpha }\left[f(x)\right]=\frac{1}{\Gamma (1-\alpha )}\int_{0}^{x}\frac{f^{\left(m+1\right)}(t)}{{\left(x-t\right)}^{\alpha }}dt,\;\;0<\alpha <$1, is applied in nonlinear reaction–diffusion equations [37].

### 3.2. The Mathematical Model of Neuronal Branch and Soma Formation

#### 3.2.1. Neuronal Branch and Soma Formation Based on Actin Cytoskeleton

Here, we primarily discuss mathematical models related to the definition of cell and branch formation, utilizing the framework of the cell cytoskeleton due to its major contribution to cell and branch formation. By the end of Section 1, we highlight how incorporating neurobiological complexity enriches mathematical and geometrical modeling of cell and branch formation, leading to a more comprehensive understanding of these processes, since we need to define mathematically the electrical activity of the cell in combination with actin cytoskeletal to be able to demonstrate a cell with electrical property that takes the cell shape. Finally, we will demonstrate that these cells can mathematically develop into an organoid brain.

**Figure 2:**
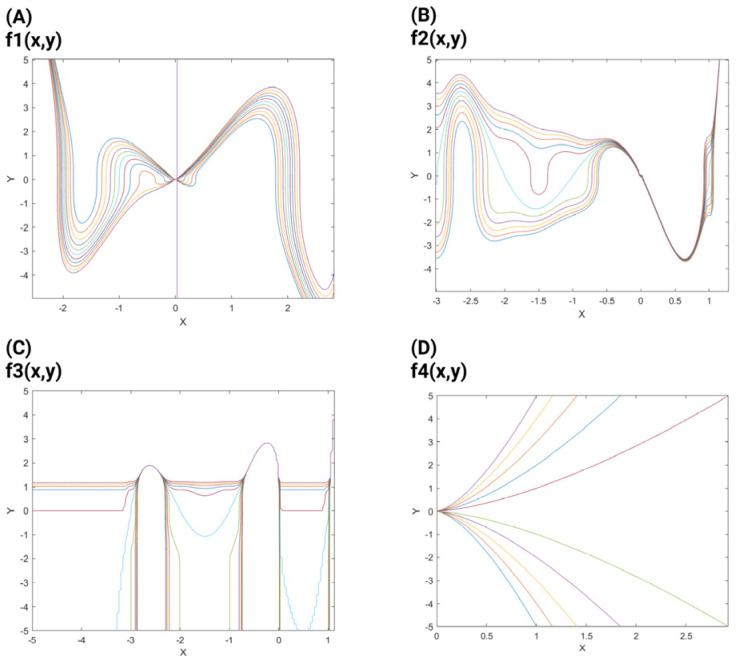
The plots demonstrate the ramifications and branching examples in mathematical terms. $A:f_{1}\left(x,y\right)=c\;-$ $\frac{y^{3}}{x^{3}}+x\left(x+1\right)$(x−2)$\left(x+2\right)(x-3)$; $B:f_{2}\left(x,y\right)=c-\frac{y^{3}}{x^{2}}+x{\left(x-1\right)}^{3}{\left(x+1\right)}^{3}{\left(x+2\right)}^{3}{\left(x+3\right)}^{3}$; $C:f_{3}\left(x,y\right)=c+e^{x{\left(x-1\right)}^{3}{\left(x+1\right)}^{3}{\left(x+2\right)}^{3}{\left(x+3\right)}^{3}}-e^{y^{3}}$;$\;D:f_{4}\left(x,y\right)=x-{\left(\frac{y}{c}\right)}^{2/3}.$ In this image, the different colors used in each subplot (**A**–**D**) represent different contour levels for the functions f(x,y). These contours correspond to different constant values of c, and each colored curve is a level curve (or contour line) where the function equals a particular constant value. The color transitions illustrate how the function changes with respect to x and y, helping to visualize the topology and branching behaviour of the function.

**Figure 3:**
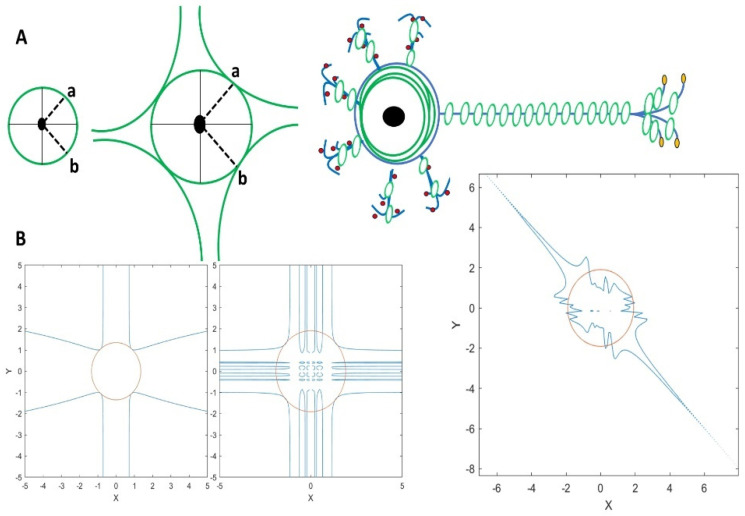
The corresponding plots related to Equation (1) are presented here. (**A**) Schematic illustration of the soma showing the two points on the boundary of the cell, a = (x + iy), b = (x − iy), which grow to the next step with four branches and later develop with the actin rings in the axons and dendrites. Dash lines connect center of the cell soma to the boundry of cell. The actin circles (green circles) are also located in the soma beneath the cell membrane. (**B**) The red circle denotes the soma defined by the Equation (1d); the Equation (1a–c) corresponds (from left to right) to the generated plots that show how geometrically neuronal branches (blue lines) can grow and develop on the basis of the characteristic polynomials that are represented as primary matrices in Equation (1a–c), respectively.

**Figure 4:**
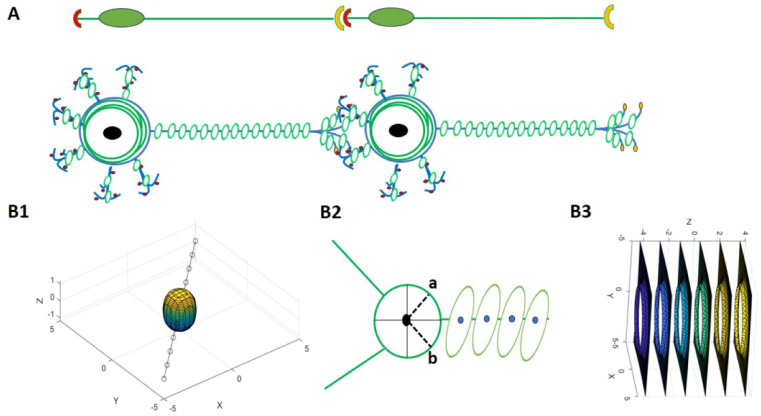
Schematic illustration of two neurons connecting with their branches containing actin rings, including the details of neuronal and actin cytoskeletal structure. (**A**) This illustration demonstrates the role of the actin ring in inter-neuronal connectivity through the formation of dendrites and axonal branches. (**B1**) The points on the line indicate the sequential actin rings along the dendrite, axonal branches, and the sphere generated according to Equation (2). (**B2**) It schematically shows the neuronal soma with two complex points: (a) z1 = (x + y1i) and (b) z2 = (x − y1i), as well as the schematic actin rings (in green) next to the main circle which is a soma containg two points of a and b on the boundary. (**B3**) It represents the cross-section of the actin rings, as per (**B3**), which is a 3D density based on Equations (3) and (4). The color spectrum (dark to bright) reflects the field intesnsity across the actin ring structure.

Mathematically, branching and ramification refer to the divergence of lines or paths from a common origin, such as the roots of algebraic equations [18]. While Vandermonde determinants do not directly represent ramification, they appear in related contexts, such as polynomial discriminants, which can indicate ramified or degenerate roots [19]. This is analogous to the neuronal branching pattern in which axons and dendrites develop from the neuronal soma, and synapse formation occurs. The mathematical equations and examples illustrating branching and ramification, along with the relevant plots, are shown in Figure 2. The circular actin cytoskeleton is also located in the neuronal soma Figure 3A. The inter-neuronal connectivity [38] is defined by the synaptic connection between the dendritic spine and axonal buttons Figure 4A. Thus, we begin to explore these anatomical elements in a stepwise manner, defining them in an illustrative way while suggesting equations that can approximate these anatomical and geometrical elements separately. Therefore, the mathematical description of branch formation is speculated through the following steps. We applied the Möbius transformation, which is denoted by the coefficient of the matrix $\left[\begin{array}{cc} a & b\\ c & d \end{array}\right]$ and is the rational function of $f\left(z\right)=\frac{az+b}{cz+d}$ [39], in which ‘*z*’ is a complex value. The circles and spheres are generated via applying Equation (1) with respect to the ring polynomial, which is denoted by B = K[X] [20], and the homogeneous polynomial [21], which are explained elsewhere.

Thus, we can apply the characteristic determinant, which is called the characteristic polynomial [22] and is denoted by $A=\left[\begin{array}{cc} a & b\\ d & c \end{array}\right],C=det\left(zI_{n}-A\right)$ that applied to the matrices, as shown here in Equation (1). The matrices of $A_{1:3},\;B$ and their correspondent $C_{1:3},\;C_{0}$ are characteristic polynomial.

(1a) $$\left(\begin{array}{cc} \frac{1}{x^{2}}+\frac{1i}{y^{2}} & 1\\ 1 & \frac{1}{x^{2}}-\frac{1i}{y^{2}} \end{array}\right)\;C_{1}\left(x,y,z\right)=\left(\frac{1}{x^{2}}-\frac{1i}{y^{2}}\right)\left(\frac{1}{x^{2}}+\frac{1i}{y^{2}}\right)-\frac{2z}{x^{2}}+z^{2}-1$$

(1b) $$\left(\begin{array}{cc} e^{\sin\left(\frac{1}{x^{2}}\right)+\frac{1i}{y^{2}}} & 1\\ 1 & e^{\sin(\frac{1}{x^{2}})-\frac{1i}{y^{2}}} \end{array}\right)\;C_{2}\left(x,y,z\right)=z^{2}+\left(-e^{\sin\left(\frac{1}{x^{2}}\right)+\frac{1i}{y^{2}}}-e^{\sin\left(\frac{1}{x^{2}}\right)-\frac{1i}{y^{2}}}\right)\;z+e^{2\sin\left(\frac{1}{x^{2}}\right)}-1$$

(1c) $$\left(\begin{array}{cc} e^{\sin\left(\frac{1}{x^{2}}\right)+\frac{1i}{y^{2}}} & x+y\\ x+y & e^{\sin(\frac{1}{x^{2}})-\frac{1i}{y^{2}}} \end{array}\right)\;C_{3}\left(x,y,z\right)=z^{2}+\left(-e^{\sin\left(\frac{1}{x^{2}}\right)+\frac{1i}{y^{2}}}-e^{\sin\left(\frac{1}{x^{2}}\right)-\frac{1i}{y^{2}}}\right)\;z+{{\left(x+y\right)}^{2}+e}^{2\sin\left(\frac{1}{x^{2}}\right)}$$

(1d) $$\left(\begin{array}{cc} x+yi & 1\\ 1 & x-yi \end{array}\right)\;C_{0}\left(x,y,z\right)=z^{2}-2xz+\left(x-yi\right)\left(x+yi\right)-1$$

The mathematical demonstration of Equation (1) is shown in Figure 3B. The circle in Figure 3B represents the soma in 2D (z = 1 was considered) by applying Equation (1d), while the neuronal branches that can grow according to the characteristic polynomials are plotted using primary matrices in Equation (1a–c), respectively.

Next, we shed light on the distribution of actin rings along the branches and explored how these rings could be formed geometrically. Thus, as shown in Figure 4B1, the points on the line indicate the actin rings that are located sequentially, and the sphere represents the cell soma, which is designed according to Equation (2).

(2) $$S=\left(x-y1i\right)(x+y1i)(-x+y1i)(-x-y1i)+z^{2}-1$$

Thus, the complex plane in Figure 4B2 shows two complex points, $z_{1}=\left(x+y1i\right)$ and $z_{2}=\left(x-y1i\right)$, indicated as a and b, respectively. So, to explore how the actin rings in the soma are connected geometrically to the actin rings in the branches, if we simply multiply the $z_{1}*z_{2}=x^{2}+y^{2}$, (x, y, z are spatial coordinates) and then if it is divided by sin (z) that reflects the sinusoidal distribution of the actin rings along the branches, so we chose a random number k = 4 in sin (4z) just to show the ring formation clearly in 3D based on mathematical equation that shown in Figure 4B1, however, this can be further clarified by knowing the actin ring periodicity, if we consider $\lambda =\frac{2\pi }{k},\;\;k=\frac{2\pi }{\lambda }$, since the value of λ reported in a study explored by [40], so regarding this value λ = 190 nm, which is related to the actin ring periodicity in the axons that could be lower in the dendrite branches and spines, therefore we obtain $k=\frac{2\pi }{190\times 10^{-9}}=\frac{3.31\times 10^{7}}{10^{6}}=33.1$ rad/μm. So, we obtain:

(3) $$h\left(x,y,z\right)=\frac{x^{2}}{sin(4z)}+\frac{y^{2}}{sin(4z)}-4;$$

Then, we can integrate over the *z-direction* along the branches, which yields:

(4) $$H\left(x,y,z\right)=\int h\left(x,y,z\right)\;dz=\ln\left(\tan\left(2z\right)\right)\left(\frac{x^{2}}{4}+\frac{y^{2}}{4}\right)-2\mathrm{atan}\;(\frac{8\tan\left(2z\right)\left(2\;x^{2}+{2\;y}^{2}\right)-64}{16\;x^{2}+16\;y^{2}-64\;tan(2z)});$$

Thus, we defined the actin circles shown in Figure 4B3 according to the aforementioned steps outlined in Equation (4). Therefore, this verifies that the actin cytoskeleton governs not only the cell soma shape formation but also has a deterministic role in branch formation, which we demonstrate mathematically. This example illustrates how actin in the cell soma, as part of the cytoskeleton, can transform into ring-like structures and develop into branches. However, the role of electrical activity in neurons, in conjunction with these structures (actin rings), in forming the main cellular structures will be presented next.

#### 3.2.2. Role of Neuronal Electrical Activity in Conjunction with the Actin Cytoskeleton in Neuronal Branch and Soma Formation

We design a fully deterministic model, based on the implementation of algebraic and geometric equations that govern the system’s structure. As such, it does not rely on stochastic elements or random initialization, and the output for a given set of input parameters is entirely reproducible. Hence, neuronal electrical activity can be elucidated from another perspective, known as the limit cycle, which is defined by trajectories in a 2D phase space and behaves as a type of oscillator [41]. The well-known oscillator is the Van der Pol equation; the “m” acts as the parameter that controls the strength of damping and the nature of oscillations:

(5) $$r=\sqrt{x^{2}+y^{2}},\;{\vec{w}}_{X2}=\frac{y}{r},\;{\vec{w}}_{Y2}=\frac{-(x+m\;y\;\left(x^{2}-1\right))}{r}$$

**Figure 5:**
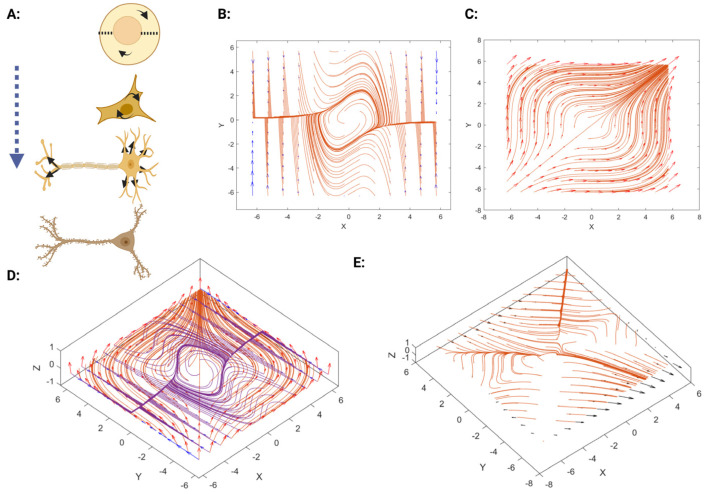
(**A**) A schematic illustration of the electrochemical interactions between the cell membrane and nuclear membrane is shown in the form of a vector plot. (**A**) The alteration in neuronal morphology and geometry is partly due to electrochemical interactions and partly due to the electromechanical effect that leads to branching and ramification in neurons. (**B**) The Van der Pol oscillator is illustrated in the 2D vector plot. Red-Orange streamlines: Vector field flow direction and intensity from the Van der Pol oscillator. Blue arrows (vertical edges): represent boundary conditions or external perturbations. (**C**) A form of concentric vector field is shown that represents the circular actin geometry inside the cell soma in 2D. Red-orange streamlines and arrows: A radial or swirling inward/outward vector field, likely illustrating actin dynamics/electrochemical patterns around the nucleus/soma. (**D**) The superimposed form of the Van der Pol oscillator and concentric vector field to compare with the next plot in. This blend reflects a superimposition of the Van der Pol (in Purple streamlines) and the circular field (in Orange streamlines) and the Red arrows: Represent vector directions. (**E**), in which their interaction was calculated. (**E**) The interaction of two vector fields by applying the iterated Lie bracket leads to nerve endings at the periphery of the vector field and branch formation. Orange: Field vectors resulting from the interaction (combined electrochemical + mechanical effects). Black arrows: Shows emergent structures, such as nerve terminals or branch tips. This highlights how interactions cause localized morphogenesis.

Which represents the electrical activity of a neuron, namely, neuronal excitability. The van der Pol oscillator is a variant of the FitzHugh–Nagumo model, which is a modified version of the Hodgkin–Huxley model [25]. The electrical activity of the cell membrane is well-known. However, electrophysiological activity in the nuclear membrane results in “core–shell models” [42]. Hence, it is a biophysical representation of a cell that captures the complex electrical and electromechanical interactions between its outer membrane (cell membrane, CM) and inner boundary (nuclear envelope, NE), so voltage difference between the exterior and interior of a cell is dependent on the interaction of the ion channels in the CM that maintains electric charge. This is especially useful for studying how electrical and electromechanical changes outside and inside influence cell shape, based on this special model. Hence, we proposed a model that incorporates two vector fields. One of these vector fields is based on the electrical activity of ion pumps and channels, which is reflected by the van der Pol equation. Meanwhile, there is another mechanism in which ion waves are transmitted along the actin cytoskeleton, and ion-dependent actin polymerization occurs as well [43]. It is demonstrated in Figure 5A. Since the patterns of spiking are different in cortical neurons and correlate with neuronal morphology [44], we mathematically suppose that, due to the diverse neuronal shapes observed in the CNS with various resting membrane potentials that are based on ${Na}^{+}$ and ${Cl}^{-}$ which range from −75 to −40 mV [45], there must be another type of vector field that exists in the cell related to the actin circle geometry beneath the cell membrane shown in Figure 4A, which contributes to this neuronal shape and, consequently, membrane potential diversity. Additionally, experimental examples have demonstrated this relationship, based on the specific fast-spiking Kv3 potassium channel found in GABAergic interneurons and Purkinje cells. Another similar example is Nav1.6 Voltage-Gated sodium channel [46]. Therefore, we can explore the interaction of these two vector fields [X, Y] = XY − YX, including van der Pol Figure 5B and the circular actin geometry Figure 5C that is located beneath the cell membrane shown in Figure 3A. It could be defined in the form of a vector field as below:

(6) $$r=\sqrt{x^{2}+y^{2}},{\vec{w}}_{X1}=\frac{y^{2}}{r},\;{\vec{w}}_{Y1}=\frac{x^{2}}{r},$$

These coexist with the Van der Pol oscillations, as shown in Figure 5D. Finally, regarding Frobenius’ theorem and the Lie bracket of vector fields that were explained elsewhere [24], we explore the interaction of these two vector fields, [X, Y] = XY − YX. However, we need to explore how one vector field affects the other, which includes the Van der Pol and circular actin geometry, first in a simple 2D model that is demonstrated in Figure 5E. The simple solution is to apply $X\left(Y\left(f\right)\right)-Y\left(X\left(f\right)\right)=\sum_{i,j=1}^{n}\left(X_{i}\frac{\partial }{\partial x_{i}}\left(Y_{j}\right)-Y_{i}\frac{\partial }{\partial x_{i}}\left(X_{j}\right)\frac{\partial }{\partial x_{j}}\left(f\right)\right),$ and for the iterated Lie bracket, we apply Equation (7) using the Jacobian. Each iteration captures a deeper level of interaction between f and g, where f and g are vector fields that receive values from Equations (5) and (6). This process is fully expressed in Equation (11), which is defined in 3D. Lie brackets can model interactions such as feedback loops between electrical fields and ionic flux:

(7) $${ad}_{f}^{\left(k\right)}g=J_{\left({ad}_{f}^{\left(k-1\right)}g\right)}.f-J_{f}\left({ad}_{f}^{\left(k-1\right)}g\right),\;J=\mathrm{Jacobian}\;\mathrm{matrix}$$

Here are examples of iterations:

$${ad}_{f}^{\left(1\right)}g=[f,g],\;k=1;$$

$${ad}_{f}^{\left(2\right)}g=[f,[f,g]],\;k=2;$$

$${ad}_{f}^{\left(3\right)}g=[f[f,[f,g]]],\;k=3;$$

Next, for this model, the integral of the values ($\iint F\left(x,y\right)\;dxdy$) obtained from Equation (7).

Then, we aimed to create a 3D mathematical model of neuronal activity based on the aforementioned vector fields. Thus, Equations (5)–(7) can be transformed into 3D equivalence Equations (8)–(10).

(8) $$r=\sqrt{x^{2}+y^{2}+z^{2}},\;{\;\vec{v}}_{X1}=\frac{y^{2}}{rz^{2}},\;{\;\vec{v}}_{Y1}=\frac{x^{2}}{rz^{2}}\Rightarrow \vec{f}\left(x,y,z\right)={\vec{v}}_{X1}\hat{l}+{\vec{v}}_{Y1\;}\hat{J}$$

(9) $${\vec{v}}_{X2}=\frac{y}{r\;z},{\vec{v}}_{Y2}=-\frac{\frac{x}{z}+\frac{m\;y\;\left(\frac{x^{2}}{z^{2}}-1\right)}{z}}{r};\;m=(0.1:4)\Rightarrow \vec{g}\left(x,y,z\right)={\vec{v}}_{X2}\hat{l}+{\vec{v}}_{Y2\;}\hat{J}$$

Hence, the *membrane potential* could be calculated according to the Goldman equation [26], which is expressed by $\int_{0}^{x}e^{\frac{FV(x)}{RT}}dx$, and the Nernst equation, which is denoted by 61.5 mV × log($\frac{x_{out}}{x_{in}}$), 61.5 mV=2.3026×26.7, and $\frac{RT}{F}=26.7\;mV$ at body temperature, which has been explained elsewhere [27]. Similarly, after obtaining the result from the iterated Lie bracket by using the Jacobian Equation (10).

(10) $${ad}_{f}^{\left(k\right)}g=J_{\left({ad}_{f}^{\left(k-1\right)}g\right)}.f-J_{f}\left({ad}_{f}^{\left(k-1\right)}g\right),\;J=\mathrm{Jacobian}\;\mathrm{matrix}$$

**Figure 6:**
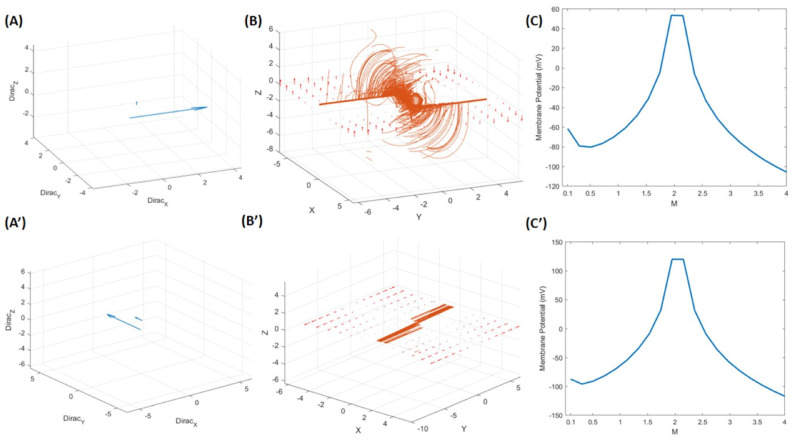
The plot represents Equation (11). (**A**) Vector direction spatially (i, j, k) when we apply it with Dirac(i, j, k)=log(e−EFTm). (**B**) Vector plot related to the gradient of the interaction of two vector fields after applying the iterated Lie bracket in 3D, similar to 2D, shown in Figure 5E. (**C**) The membrane potential was calculated by applying the electric field as described in Equation (11). The ‘M’ denotes that the van der Pol vector field changes. (**A’**) shows the Dirac in three axes if we use the vector Laplacian. ‘(**B**’) represents the vector plot related to the vector Laplacian of the interaction of two vector fields in 3D. ‘(**C**’) demonstrates the Dirac of the vector Laplacian. (**A**,**A’**): Blue direction and magnitude of Dirac vector fields; (**B**,**B’**): Orange/Red vector field interaction; flow lines & gradients; (**C**,**C’**): Blue (line) is representative of membrane potential plot.

Thus, we applied the Electric Field=−∇F [30] to obtain the *membrane potential* [47]. Alternatively, we can use Potential of Laplacian=−∇(∇.F) or Vector Laplacian=∇(∇.F)−∇×(∇×F). Then, we use the Dirac delta function [28] $\int_{-\infty }^{+\infty }\delta \left(x-a\right)f\left(x\right)=f\left(a\right)$ in form of $\int_{-\infty }^{+\infty }\delta \left(x-a\right)e^{-sx}=e^{-as}\;$ (m = (0.1:4) in Dirac that is similar to canonical structure of spatial exponential decay that was used for example for analyzing spatiotemporal wave patterns in neural activity in neural field model [48]) and finally used the Nernst equation to obtain neurobiologically adapted values. The summarized functions related to the calculation of the membrane potential are shown in Equation (11), and the corresponding plots are shown in Figure 6. “Although units are not explicitly attached to the symbolic expressions, the formulation follows established principles of dimensional analysis and non-dimensionalization [49]. This approach improves numerical behavior and highlights scale-invariant structures—core benefits of non-dimensional modeling”. However, for clarity and completeness, we provide Table 1, which lists the relevant units based on the information associated with Equation (11), our main governing equation.

(11) $$\vec{f}=\left[\begin{array}{c} {\vec{v}}_{X1}\\ {\vec{v}}_{Y1\;} \end{array}\right],\;\vec{g}=\left[\begin{array}{c} {\vec{v}}_{X2}\\ {\vec{v}}_{Y2\;} \end{array}\right]\;T\underset{\_\_\_\_\_\_\_\_\_\_\_\_\_\_\_\_\_\_\_}{\mathrm{he vector fields}}$$

$${ad}_{f}^{\left(k\right)}g=J_{\left({ad}_{f}^{\left(k-1\right)}g\right)}.f-J_{f}\left({ad}_{f}^{\left(k-1\right)}g\right):\;\underset{\_\_\_\_\_\_\_\_\_\_\_\_\_\_\_\_\_\_\_\_\_\_\_\_}{\mathrm{Iterated Lie bracket}}$$

$$Z={ad}_{f}^{\left(t\right)}g\;\to \;V=[Z_{\left(1\right)}+{\;Z}_{\left(2\right)}]:\;\underset{\_\_\_\_\_\_\_\_\_\_\_\_\_\_\_\_\_\_\_\_\_\_\_\_\_\_\_\_\_\_\_\_\_\_\_\_\_\_\_\_\_\_\_\_\_}{\mathrm{The result of the iterated Lie bracket}}$$

$$EF=-\nabla V;\;\to \;{EF}_{T}=\sqrt{{{EF}_{x}}^{2}+{{EF}_{y}}^{2}+{{EF}_{z}}^{2}}:\;\underset{\_\_\_\_\_\_\_\_\_\_\_\_\_\_\_\_\_\_\_\_\_\_\_\_\_\_\_\_\_\_\_\_\_\_\_\_\_\_\_}{\mathrm{Total electric field in three axes}}$$

∫−∞+∞δx−ae−sx=e−as →Dirac=log(e−EFTm): Dirac delta function_________________________

$$MB=log\left(Dirac\right)*61.5:\;\underset{\_\_\_\_\_\_\_\_\_\_\_\_\_\_\_\_\_\_\_\_\_\_\_\_\_\_\_\_\_\_\_\_\_\_\_\_\_\_\_\_\_\_\_\_\_\_\_\_\_\_\_\_\_\_\_\_\_\_\_}{\mathrm{Nernst equation and membrane potential (MB)}}$$

Furthermore, we decided to transform this recent mathematical model, which represents a single neuron with electrical activity similar to neuronal membrane potential, into an ensemble of neurons. Thus, we aimed to transform this equation such that a single neuron achieves the potential to divide and grow as an ensemble of neurons, yielding an organoid brain-like structure [50]. The resemblance of the organoid brain to our model is due to the dense, radially organized, and multi-branched 3D configurations of our model, which make it morphologically equivalent to the brain organoids. The vector fields and potential gradients of our model demonstrate signal propagation in organoid networks that closely match the electrophysiological dynamics of real organoids, providing a mathematical analogy. Furthermore the human brain organoid is a developed 3D brain tissue derived from pluripotent stem cells that they simply show the spikes action potential that are “self-patterning systems” which produce various cellular structure and form the endogenous tissue [51], for example, the resting membrane potential derived from spiny neurons from rodents in a study was recoded around −78 mV, [52]. In another study, these 3D neural tissues (organoid tissue) exhibited variations in both the frequency and amplitude of action potential depending on the neuronal maturity [53]. Therefore, our model resembles it from two aspects: structurally, it is analogous, and the electrical activity has a similar range with multiple spikes.

**Figure 7:**
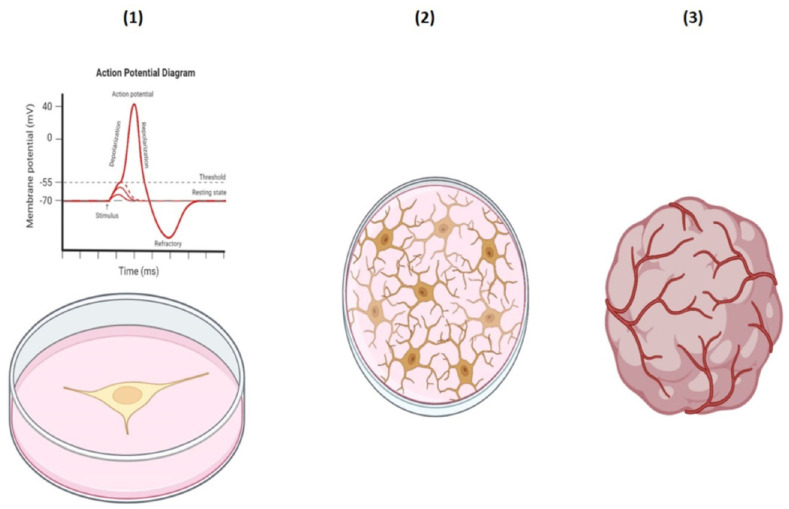
A schematic illustration of a single neuron with recorded electrical activity and action potential (**1**) **left** side is representative of Figure 6. However, the schematic illustration of cultured neurons (**2**) and an organoid brain (**3**) on the **right** side shows the equivalent mathematical models demonstrated in Figure 8.

**Figure 8:**
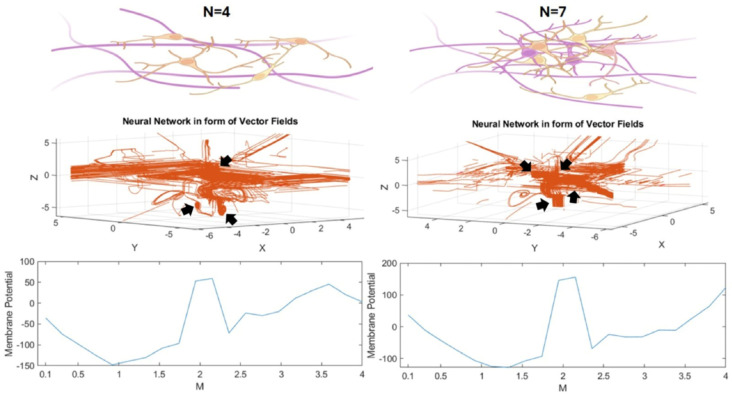
The neural network in the form of a mathematical model with its relevant membrane potential (the blue curve). This design looks like a bunch of neurons in the form of a 3D cell culture (like cultured neurons or an organoid brain, red network); so, N = 4 neurons generated on the left side, and the right side is generated by N = 7 with comparatively stronger action potential tends to become more like an organoid brain. The black arrows indicate visible node-like neurons.

The schematic neuronal culture and organoid brain are shown in Figure 7; equivalently, the mathematical model by applying the Equations (12)–(16) represents this neural network, which is provided in form of a 3D network of neurons with the ability to grow and develop like an organoid brain, as shown in Figure 8. Interestingly, the corresponding action potential of this 3D neural network consists of several peaks, implying the existence of multiple neuronal electrical activities. Additionally, it is necessary to further clarify that the spikes of the action potential shown in Figure 8 were generated based on a mathematically derived scalar field variation, which is a coupling of geometry and oscillator projected along selected spatial paths. As such, in contrast to biophysical simulations (e.g., Hodgkin–Huxley), these spikes and waveforms were not expected to match the electrophysiological recordings exactly, but instead to reflect the spatial variation in a potential function across the neural network structure. Hence, the multiple peaks represent spatial, localized potential variations in the constructed neural network. However, by applying the Nernst equation, the action potential was able to reflect neurophysiologically adapted values.

The mathematical transformation process for generating a 3D neural network is indicated by parameters shown in Equations (12)–(16). First, it includes ‘*V*’, which implies the interaction of the actin filaments and van der Pol oscillator in the form of vector fields by using the Lie bracket; this step is similar to single neuron formation, and the membrane potential was calculated according to Equation (11).

(12) $$V=\frac{y}{z\;\sqrt{x^{2}+y^{2}+z^{2}}}-\frac{x}{z\;\sqrt{x^{2}+y^{2}+z^{2}}}+\frac{y\;\bar{m}}{z\;\sqrt{x^{2}+y^{2}+z^{2}}}-\frac{x^{2}y\;\bar{m}}{z^{3}\;\sqrt{x^{2}+y^{2}+z^{2}}}$$

Next, we attempt to transform ‘*V*’ such that to guide the growing neurons spatially by applying the ‘*P*(*x*, *y*, *z*)’ as a 3D numerical map, such that it introduces curvature that affects the growth of neurons in the simulation, Equations (13)–(15).

(13) $$P(x,y,z)=-x^{3}z^{3}-a\;x^{2}z+x+y^{2}z^{2}-b$$

(14) $$F_{x}=\frac{\partial P}{\partial x}+V;\;F_{y}=\frac{\partial P}{\partial y}+V;\;F_{z}=\frac{\partial P}{\partial z}+V$$

(15) $$G_{x}=\int (\frac{\partial P}{\partial x}+V)dx;\;G_{y}=\int (\frac{\partial P}{\partial y}+V)dy;\;G_{z}=\int (\frac{\partial P}{\partial z}+V)dz$$

Further, Lx, Ly, and Lz represent the inverses of the Vandermonde matrix with Lagrange interpolation [33]. The number of neurons that grow in this 3D neural network is determined by ‘N’.

In summary, Equation (12) defines a vector field “V” that couples actin filament-incorporated geometry with the Van der Pol oscillator using Lie brackets. This provides a localized directional field that mimics the initial guidance of neurites. Equations (13)–(15) describe the application of a 3D polynomial “P(x, y, z)”, and its gradients (Fx, Fy, Fz) that guide growth trajectories. The integrals Gx, Gy, and Gz represent accumulated path information for each direction. Equation (16) introduces Lagrange interpolants using a Vandermonde inverse to solve discrete node placements in the 3D space. These positions form the topological skeleton of the synthetic network shown in Figure 8. The MATLAB codes used in this study are provided.

(16) $$\begin{array}{c} L_{x}=\sum_{i=1}^{N}\frac{G_{x^{i}}}{i+x+Fx^{i}ln(-e^{\left(i-x\right)\left(i-y\right)\left(i-z\right)}(i-x))},\;L_{y}=\sum_{i=1}^{N}\frac{G_{y^{i}}}{i+y+Fy^{i}ln(-e^{\left(i-x\right)\left(i-y\right)\left(i-z\right)}(i-y))},\;\\ L_{z}=\sum_{i=1}^{N}\frac{G_{z^{i}}}{i+z+Fz^{i}ln(-e^{\left(i-x\right)\left(i-y\right)\left(i-z\right)}(i-z))} \end{array}$$

## 4. Discussion

We applied our mathematical strategies to view how neural progenitor cells evolve to form a regional network of neurons with their specific connectivity via intercellular signaling. During brain development, neuronal development is regulated by intercellular signaling through primary cilia, which influences neuronal morphology. This guides the regional neuron to connect properly in a cluster. The role of primary cilia during neurodevelopment has been explored to model the bending rigidity, which estimates the elasticity of a filament by calculating the unit tangent vector of the filament from the base to the tip [54,55]. Functional networks are necessary for regulating structural neuroplasticity, including neurogenesis, during the embryonic period. Sonic Hedgehog (Shh) signaling operates through the primary cilium [56], and mathematical modeling of Shh signaling through the primary cilium was developed by applying partial differential equations, incorporating parameters such as Shh concentration, diffusivity, and a linear degradation model. The reaction-diffusion equation [57] was used to model the signaling through the (Shh) in 2D, which is $\partial_{t}q=D\nabla^{2}q+R(q)$. It has been shown that a mathematical equation (Bessel function) $\frac{x^{2}d^{2}y}{dx^{2}}$ + $\frac{xdy}{dx}+(x^{2}-n^{2})y=0$ is applicable for modeling cellular actin, which is called the cellular cortex, behaves like a vibrating surface and can be deterministic for the cellular shape [58]. In another study, the equation of motion was applied to model F-actin growth $\frac{\partial u}{\partial t}$ + *u*.$\nabla u=\frac{-\nabla f}{P}$ which includes the following parameters: *f* = fluid density, *p* = pressure, and *u* = fluid velocity. It has been shown that the length of filaments in the actin cytoskeleton changes [59].

Next, our study demonstrated how actin filaments organize spatially in a ring-like structure along neuronal branches, contributing to branch and spine formation. They are oriented both as ring-like structures and longitudinal filaments. The mathematical model of ramification and branching for structures such as dendrites and axons associated with the neuronal soma was also expressed in terms of actin filaments. The actin cytoskeleton plays a vital role neurobiologically, as the intracellular actins near the nucleus affect cell shape and morphology [60] while regulating chromatin. They modulate the cell shape in connection with the nearest neuron by receiving external signals. Thus, these elements could influence the arrangement and organization of adjacent neurons, shaping their structures, such as proper dendrite arborization in pyramidal neurons.

Additionally, actin plays a crucial role in regulating the formation, structure, and function of synapses [61]. When expressed in mathematical terms, the role of actin filaments is employed to characterize branching and ramification in neurons [18], ultimately contributing to the development of axons and dendrites, as well as the formation of synapses. The fundamental process is associated with actin rings, which serve as protein scaffolds along neuronal branches. Following the formation of actin rings, dendritic spines emerge, and longitudinally oriented actins transform into spines, creating branched F-actin that maintains the structural integrity of the spine [6,62]. Using the polynomial ring [20], homogeneous polynomials [21], and characteristic polynomials [22], we can geometrically demonstrate the formation of circles, spheres, and ramification, which represent the neuronal soma and branches. The mathematical demonstration in Section 1 was an introduction to how demonstrate and model geometrically the neuronal branches based on mathematical equations without considering the details of the biological property of neurons such as electrical activity or specific geometrical shapes of the cytoskeleton, so one would not be biased or limited by the current biological findings while modeling mathematically, therefore it could help in improving and strengthening the mathematical knowledge, because there are variety of mathematical solutions to the apparently stochastic biological models.

Nevertheless, we investigated the involvement of the actin cytoskeleton in neuronal electrical activity via a vector field approach. This suggests a more complex form of interaction between geometrical and vector fields, demonstrated through the application of Lie algebra to 2D and 3D neuronal shapes. This reveals the interplay between the assumed concentric vector field of actin’s geometrical structures and the neuron’s electrical activity, referred to as the Van der Pol, which exhibits a limit cycle. Moreover, the definition of neuronal electrical activity was established through the limit cycle [41], incorporating the Van der Pol equation, which is associated with neuronal excitability and gives rise to “core–shell models” [42]. These models represent electrochemical interactions between the cell membrane and the nuclear membrane. The diverse spiking patterns observed in neurons are dependent on neuronal morphology [44]. In terms of the impact of electricity on the cell [47], an additional type of vector field exists within the cell, generated by the actin circle situated beneath the cell membrane, resulting in the formation of a concentric vector field that aligns with our anticipation. The interaction between these two vector fields is calculated by applying the Lie bracket and explained by Frobenius’ theorem [24]. Therefore, neuronal branching and synapse formation can be partly explained by the interaction of the vector field, as shown in Figure 5E.

Next, we generated a 3D model of neuronal electrical activity and explored whether electrical activity can be translated in the context of the neuronal membrane potential. Our model revealed that action potentials with amplitudes ranging from −60 to +55 mV are compatible with neuronal action potentials. In the CNS, the resting membrane potential ranges from −75 to −40 mV [45], and sodium channels are activated at a resting membrane potential of −65 mV [63]. In contrast, when we used the vector Laplacian to compare with the electric field (−∇*F*) equation, the same membrane potential was not obtained; it ranges from −100 to 100 mV, and the shape of the neuron’s vector field became quite different. So, it seems that the comparative electric field (−∇F) equation is more accurate and appropriate for our purpose. Comparably, the Hodgkin-Huxley model is a point neuron model, in contrast to our 3D single neuronal model, which incorporates built-in electrical activity and captures similar neuronal electrical properties. The HH model comprises four differential equations that govern the evolution of the membrane potential and the opening of ionic channels in the cell membrane. The drawback of the HH model is that it is quite complicated and very difficult to analyze mathematically in terms of a network of interacting neurons [64].

In the last step, we generate a framework of neurons that can grow and develop similarly to an organoid brain, with a certain action potential in which the voltage amplitude varies depending on the number of neurons and their spatial formation in a neuronal cluster, which resembles an organoid brain.

**Limitations:** Further study is needed to design an equation analogous to Nörrland polynomials [65] and Stirling polynomials. Our mathematical model is expected to benefit from improving the geometrical design of synapse formation in connection with neuronal electrical activity in a specific brain region with distinct neuronal clusters that incorporate various types of neurons, synchronized via their electrical activity and synapses.

**Conclusions:** We propose an equation that illustrates the relationship between the actin cytoskeleton and the signaling processes of primary cilia. We introduced a mathematical definition for neuronal branches using a geometrical ramification approach and provided a mathematical definition for neuronal soma and branch formation in two dimensions. Furthermore, we demonstrated that both neuronal electrical activity and the actin cytoskeleton influence changes in 2D neuronal morphology, and the resulting 3D model of neuronal electrical activity resembles a core-shell structure that reflects the neuronal membrane potential. Furthermore, this model can evolve into a neural network with a specific action potential that could potentially contribute to advancements in artificial intelligence and neural networks.

## Figures and Tables

**Table 1:** A summarized list of Equation (11), including its definition and corresponding units, is presented.

Symbol/Expression	Meaning	Units
x,y,z	Spatial coordinates (position)	µm (micrometers)
$r=\sqrt{x^{2}+y^{2}+z^{2}}$	Euclidean distance from origin	µm
m=(0.1:4)	Nonlinear control parameter	dimensionless
$\vec{f}\left(x,y,z\right)={\vec{v}}_{X1}\hat{l}+{\vec{v}}_{Y1\;}\hat{J}$ $\vec{g}\left(x,y,z\right)={\vec{v}}_{X2}\hat{l}+{\vec{v}}_{Y2\;}\hat{J}$	Vector Fields	mV/µm
${ad}_{f}^{\left(k\right)}g=J_{\left({ad}_{f}^{\left(k-1\right)}g\right)}.f-J_{f}\left({ad}_{f}^{\left(k-1\right)}g\right)$	Lie bracket between $\vec{f}$ and $\vec{g}$	mV/µm^2^
$Z={ad}_{f}^{\left(t\right)}g\;\;\to \;\;\;V=[Z_{\left(1\right)}+{\;Z}_{\left(2\right)}]$	Lie bracket (recursive)	mV/µm^2^
EF=−∇V	Gradient of scalar potential field	mV/µm^3^
∫−∞+∞δx−ae−sx=e−as →D =log(e−EFTm)	Activation-like potential field	dimensionless
MB=log(D)×61.5	Membrane potential derived from the field	mV

## Data Availability

All data reported in this paper will be shared by the lead contact upon request.

## References

[1] Lana D., Ugolini F., Nosi D., Wenk G.L., Giovannini M.G. (2017). Alterations in the interplay between neurons, astrocytes and microglia in the rat dentate gyrus in experimental models of neurodegeneration. Front. Aging Neurosci..

[2] Hua J.Y., Smith S.J. (2004). Neural activity and the dynamics of central nervous system development. Nat. Neurosci..

[3] Yamamoto N., López-Bendito G. (2012). Shaping brain connections through spontaneous neural activity. Eur. J. Neurosci..

[4] Visa N., Percipalle P. (2010). Nuclear functions of actin. Cold Spring Harb. Perspect. Biol..

[5] Shim S., Goyal R., Panoutsopoulos A.A., Balashova O.A., Lee D., Borodinsky L.N. (2023). Calcium dynamics at the neural cell primary cilium regulate Hedgehog signaling–dependent neurogenesis in the embryonic neural tube. Proc. Natl. Acad. Sci. USA.

[6] Konietzny A., Bär J., Mikhaylova M. (2017). Dendritic actin cytoskeleton: Structure, functions, and regulations. Front. Cell. Neurosci..

[7] Oertner T.G., Matus A. (2005). Calcium regulation of actin dynamics in dendritic spines. Cell Calcium.

[8] Kiss J.Z., Müller D. (2001). Contribution of the neural cell adhesion molecule to neuronal and synaptic plasticity. Rev. Neurosci..

[9] Drenckhahn D., Frotscher M., Kaiser H.W. (1984). Concentration of F-actin in synaptic formations of the hippocampus as visualized by staining with fluorescent phalloidin. Brain Res..

[10] Singla V., Reiter J.F. (2006). The primary cilium as the cell’s antenna: Signaling at a sensory organelle. Science.

[11] Wheway G., Nazlamova L., Hancock J.T. (2018). Signaling through the primary cilium. Front. Cell Dev. Biol..

[12] Peng T., Cheng Y., Xiong M., Zhou W.-H., Cheng G.-Q. (2022). Primary cilia in the development of the cerebral cortex: A literature. Pediatr. Med..

[13] Smith C.E., Lake A.V., Johnson C.A. (2020). Primary cilia, ciliogenesis and the actin cytoskeleton: A little less resorption, a little more actin please. Front. Cell Dev. Biol..

[14] Markram H. (2006). The blue brain project. Nat. Rev. Neurosci..

[15] Holmes W.R., Edelstein-Keshet L. (2016). A comparison of computational models of the dendritic actin cytoskeleton. Cytoskeleton.

[16] Lynn C.W., Bassett D.S. (2019). The physics of brain network structure, function and control. Nat. Rev. Phys..

[17] Rafati A.H., Joca S., Vontell R.T., Wegener G., Ardalan M. (2024). Approaches to embryonic neurodevelopment: From neural cell to neural tube formation through mathematical models. Brief. Bioinform..

[18] van Hoeij M., Vidunas R. (2018). Algorithms and differential relations for Belyi functions. arXiv.

[19] Opfer G. (2015). Polynomial interpolation in nondivision algebras. Electron. Trans. Numer. Anal..

[20] Robbiano L. Term orderings on the polynomial ring. Proceedings of the European Conference on Computer Algebra.

[21] Berele A. (1982). Homogeneous polynomial identities. Isr. J. Math..

[22] Amitsur S.A. (1980). On the characteristic polynomial of a sum of matrices. Linear Multilinear Algebra.

[23] Kaczmarek L.K., Zhang Y. (2017). Kv3 channels: Enablers of rapid firing, neurotransmitter release, and neuronal endurance. Physiol. Rev..

[24] Feleqi E., Rampazzo F. (2017). Iterated Lie brackets for nonsmooth vector fields. Nonlinear Differ. Equ. Appl. NoDEA.

[25] Franci A., Drion G., Seutin V., Sepulchre R. (2011). A novel phase portrait to understand neuronal excitability. arXiv.

[26] Barr L. (1965). Membrane potential profiles and the Goldman equation. J. Theor. Biol..

[27] Cavagna G. (2019). Fundamentals of Human Physiology.

[28] Balakrishnan V. (2003). All about the Dirac delta function (?). Resonance.

[29] Dayan P., Abbott L.F. (2005). Theoretical Neuroscience: Computational and Mathematical Modeling of Neural Systems.

[30] Griffiths D.J. (2017). Introduction to Electrodynamics.

[31] Silverman J.H., Tate J. (1992). Rational Points on Elliptic Curves.

[32] Silverman J.H. (2009). The Arithmetic of Elliptic Curves.

[33] Verde-Star L. (1988). Inverses of generalized Vandermonde matrices. J. Math. Anal. Appl..

[34] Breunig J.J., Sarkisian M.R., Arellano J.I., Morozov Y.M., Ayoub A.E., Sojitra S., Wang B., Flavell R.A., Rakic P., Town T. (2008). Primary cilia regulate hippocampal neurogenesis by mediating sonic hedgehog signaling. Proc. Natl. Acad. Sci. USA.

[35] Derrick D.J., Wolton K., Currie R.A., Tindall M.J. (2021). A mathematical model of the role of aggregation in sonic hedgehog signalling. PLoS Comput. Biol..

[36] Reinartz K.D. (2016). Chebychev interpolations of the Gamma and Polygamma Functions and their analytical properties. arXiv.

[37] Owolabi K.M. (2017). Mathematical modelling and analysis of two-component system with Caputo fractional derivative order. Chaos Solitons Fractals.

[38] Stepanyants A., Hof P.R., Chklovskii D.B. (2002). Geometry and structural plasticity of synaptic connectivity. Neuron.

[39] Arnold D.N., Rogness J.P. (2008). Möbius transformations revealed. Not. Am. Math. Soc..

[40] Xu K., Zhong G., Zhuang X. (2013). Actin, spectrin, and associated proteins form a periodic cytoskeletal structure in axons. Science.

[41] Giné J. (2012). Limit cycle bifurcations from a non-degenerate center. Appl. Math. Comput..

[42] Sabri E., Brosseau C. (2024). Electromechanical interactions between cell membrane and nuclear envelope: Beyond the standard Schwan’s model of biological cells. Bioelectrochemistry.

[43] Tuszyński J.A., Portet S., Dixon J.M., Luxford C., Cantiello H.F. (2004). Ionic Wave Propagation along Actin Filaments. Biophys. J..

[44] Connors B.W., Gutnick M.J. (1990). Intrinsic firing patterns of diverse neocortical neurons. Trends Neurosci..

[45] McCormick D.A. (2014). Chapter 12-Membrane Potential and Action Potential. From Molecules to Networks.

[46] Akin E.J., Solé L., Dib-Hajj S.D., Waxman S.G., Tamkun M.M. (2015). Preferential targeting of Nav1. 6 voltage-gated Na^+^ channels to the axon initial segment during development. PLoS ONE.

[47] Kotnik T., Miklavčič D. (2000). Analytical description of transmembrane voltage induced by electric fields on spheroidal cells. Biophys. J..

[48] Shamsara E., Yamakou M.E., Atay F.M., Jost J. (2024). Dynamics of neural fields with exponential temporal kernel. Theory Biosci..

[49] Lin C.-C., Segel L.A. (1988). Mathematics Applied to Deterministic Problems in the Natural Sciences.

[50] Marton R.M., Pașca S.P. (2020). Organoid and assembloid technologies for investigating cellular crosstalk in human brain development and disease. Trends Cell Biol..

[51] Quadrato G., Nguyen T., Macosko E.Z., Sherwood J.L., Min Yang S., Berger D.R., Maria N., Scholvin J., Goldman M., Kinney J.P. (2017). Cell diversity and network dynamics in photosensitive human brain organoids. Nature.

[52] Zourray C., Kurian M.A., Barral S., Lignani G. (2022). Electrophysiological properties of human cortical organoids: Current state of the art and future directions. Front. Mol. Neurosci..

[53] Kang R., Park S., Shin S., Bak G., Park J.-C. (2024). Electrophysiological insights with brain organoid models: A brief review. BMB Rep..

[54] Kirschen G.W., Xiong Q. (2017). Primary cilia as a novel horizon between neuron and environment. Neural Regen. Res..

[55] Berbari N.F., O’Connor A.K., Haycraft C.J., Yoder B.K. (2009). The primary cilium as a complex signaling center. Curr. Biol..

[56] Anvarian Z., Mykytyn K., Mukhopadhyay S., Pedersen L.B., Christensen S.T. (2019). Cellular signalling by primary cilia in development, organ function and disease. Nat. Rev. Nephrol..

[57] Kaltenbacher B., Rundell W. (2020). Recovery of multiple coefficients in a reaction-diffusion equation. J. Math. Anal. Appl..

[58] Moore P., Huxley H., DeRosier D. (1970). Three-dimensional reconstruction of F-actin, thin filaments and decorated thin filaments. J. Mol. Biol..

[59] Hu J., Othmer H.G. (2011). A theoretical analysis of filament length fluctuations in actin and other polymers. J. Math. Biol..

[60] e Silva M.S., Alvarado J., Nguyen J., Georgoulia N., Mulder B.M., Koenderink G.H. (2011). Self-organized patterns of actin filaments in cell-sized confinement. Soft Matter.

[61] Gentile J.E., Carrizales M.G., Koleske A.J. (2022). Control of Synapse Structure and Function by Actin and Its Regulators. Cells.

[62] Nechipurenko I.V., Doroquez D.B., Sengupta P. (2013). Primary cilia and dendritic spines: Different but similar signaling compartments. Mol. Cells.

[63] Kress G.J., Mennerick S. (2009). Action potential initiation and propagation: Upstream influences on neurotransmission. Neuroscience.

[64] Li S., Liu N., Zhang X., McLaughlin D.W., Zhou D., Cai D. (2019). Dendritic computations captured by an effective point neuron model. Proc. Natl. Acad. Sci. USA.

[65] Ward M.D. (2012). Asymptotic analysis of the Nörlund and Stirling polynomials. Appl. Anal. Discret. Math..

